# The Nrf2-HO-1 system and inflammaging

**DOI:** 10.3389/fimmu.2024.1457010

**Published:** 2024-09-24

**Authors:** Sinead A. O’Rourke, Lianne C. Shanley, Aisling Dunne

**Affiliations:** ^1^ School of Biochemistry and Immunology, Trinity College Dublin, Dublin, Ireland; ^2^ Centre for Advanced Material and Bioengineering Research (AMBER), Trinity College Dublin and Royal College of Surgeons in Ireland, Dublin, Ireland; ^3^ School of Medicine, Trinity College Dublin, Dublin, Ireland

**Keywords:** inflammaging, oxidative stress, Nrf2, heme oxygenase, aging

## Abstract

Nrf2 is a master transcriptional regulator of a number of genes involved in the adaptive response to oxidative stress. Among the genes upregulated by Nrf2, heme oxygenase-1 (HO-1) has received significant attention, given that the products of HO-1-induced heme catabolism have well established antioxidant and anti-inflammatory properties. This is evidenced in numerous models of inflammatory and autoimmune disease whereby induction of HO-1 expression or administration of tolerable amounts of HO-1 reaction products can ameliorate disease symptoms. Unsurprisingly, Nrf2 and HO-1 are now considered viable drug targets for a number of conditions. In recent years, the term ‘inflammaging’ has been used to describe the low-grade chronic inflammation observed in aging/aged cells. Increased oxidative stress is also a key factor associated with aging and there is convincing evidence that Nrf2, not only declines with age, but that Nrf2 and HO-1 can reduce cellular senescence and the senescence-associated secretory phenotype (SASP) which is now considered an underlying driver of age-related inflammatory disease. In this review, we describe the role of oxidative stress in ‘inflammaging’ and highlight the potential anti-aging properties of the Nrf2-HO-1 system. We also highlight established and newly emerging Nrf2 activators and their therapeutic application in age-related disease.

## Introduction

According to demographics published by the World Health Organization, by the year 2050, the number of individuals over the age of 60 is expected to grow to 2 billion worldwide (1, 2). While this reflects a positive development in society, representing many successful endeavors to combat life-threatening diseases, we are now faced with the growing issue of chronic age-related diseases and co-morbidities. One of the major challenges that occurs in the ageing process is dysregulation of the immune response (3–6). Under normal homeostatic conditions, activation of the immune system following infection or traumatic injury, is a tightly regulated and temporal process. However, in ageing individuals, immune cell function becomes altered leading to a chronic inflammatory state. The condition, referred to as “inflammaging,” is characterized by the presence of high circulating levels of pro-inflammatory cytokines such as IL-1, IL-6, IL-8, C-reactive protein, IFN proteins α and β, and TNF (7, 8). The persistent presence of low-grade inflammation has detrimental effects on tissue homeostasis, promoting sustained activation of circulating immune cells and impairing processes such as tissue repair and effective surveillance against potential invading pathogens (9–12). As a result, older individuals have a higher risk of developing a number of chronic immune-mediated diseases (6, 7, 13). In this review, we discuss drivers of inflammaging with particular focus on oxidative stress as well as the protective and potential ‘anti-inflammaging’ effects of the Nrf2 HO-1 system.

## Drivers of inflammaging

Numerous factors are thought to contribute to inflammaging, including genetic predisposition, changes in metabolic function of immune cells, changes to the gut microbiome, chronic infections (e.g. HIV) and lifestyle habits (e.g. diet, smoking etc) (8). In particular, there is significant discussion regarding the role of senescent cells and their corresponding senescence associated secretory phenotype (SASP) (14). Induction of a senescent phenotype in immune cells, now referred to as ‘immunosenescence’, occurs in part due to the accumulation of DNA breaks following exposure to increased levels of reactive oxygen species (ROS). Senescent cells undergo epigenetic alterations, simulate chronic antigen stimulation and in turn secrete a number of soluble factors including matrix metalloproteases and growth factors as well as pro-inflammatory cytokines and chemokines (15). Both *in vivo* and *in vitro* models of ageing reveal SASP-mediated activation of immune cells, characterized by increased infiltration of neutrophils, monocytes and macrophages, NK cells, and T-cells, as well as corresponding expression of pro-inflammatory surface markers and cytokines (16, 17). Senescent immune cells display a significant impairment of function and regulation, thus further contributing to the chronic inflammatory environment observed in inflammaging (6). In support of this, increased numbers of senescent cells have been observed in individuals presenting with age-related diseases, while removal of senescent cells has recently been shown to attenuate inflammation and improve physical function in aged mice (18). This suggests that immunosenescence is a major risk factor for age-related disease and that targeting senescent cells could represent a potential strategy to treat age-related illness and inflammaging.

Damage-associated molecular patterns (DAMPs) have also been implicated in inflammaging, given their accumulation in older individuals, as well as their established role in inflammation (19). Typical DAMPs include the by-products of necrotic cells, extracellular ATP, uric acid, amyloid fibrils, misfolded/modified proteins, atherosclerosis-associated cholesterol crystals and osteoarthritis (OA)-associated basic calcium phosphate crystals, all of which are known to drive inflammation via activation of innate immune cells (20). Similar to senescent cells, the accumulation of DAMPs is associated with a number of age-related pathologies. For example, in cardiovascular disease (CVD), cholesterol crystals play a significant role in atherogenesis via recruitment and activation of inflammatory macrophages (21, 22). In Alzheimer’s disease, accumulation of extracellular amyloid beta in senile plaques is thought to be a significant contributor to disease progression, promoting sterile inflammation via activation of the NLRP3 inflammasome (23). Similarly in aged related macular degeneration, the most common cause of blindness in older individuals, disease progression is in part attributed to NLRP3-mediated inflammation driven by the accumulation of drusen deposits in the retina (24). DAMP-mediated inflammation is also thought to be further exacerbated in older individuals, due to impaired autophagy which is otherwise responsible for efficient removal and recycling of cell debris (25).

## Oxidative stress and inflammaging

Arguably, the largest contributor to inflammaging is oxidative stress, which not only promotes inflammation through the generation of DAMPs, senescent cells and activation of inflammatory signaling pathways, but also creates a relentless and destructive cycle as inflammation itself results in the production of ROS and thus further oxidative stress (26). Excessive levels of ROS generate DAMPs through oxidation and alteration of various proteins (via modification of cysteine residues), and nucleic acids (via DNA damage) leading to the production of abnormal or misfolded proteins. Oxidation of lipids by ROS forms lipid peroxidases such as malondialdehyde (MDA) and 4-hydroxy-2-nonenal (HNE) which further contribute to protein misfolding given their high affinity for the NH_2_ groups of proteins. These abnormalities ultimately disrupt normal cell function and eventually lead to cell death, whereby the abnormal proteins are released from the rupturing cells as DAMPs, along with oxidized lipids and other damage-associated cell debris (27).

ROS also contribute to cellular senescence through several mechanisms including disruption of mitochondrial function (either through direct damage of mitochondrial DNA (mtDNA) or through damage to the mitochondrial membrane via perioxidation of membrane phospholipids), inhibition of autophagy (mediated by activation of p53 signaling) and interactions with telomere regulatory enzymes such as telomere reverse transcriptase (TERT) (28–30). The combined effect of these interactions drives the onset of cell senescence, and production of the accompanying SASP. As well as contributing to the generation of DAMPs and senescent cells, ROS can also activate inflammatory signaling pathways such as those leading to NF-κB activation (31, 32). For example, high levels of ROS can activate the PI3K/AKT pathway which in turn promotes phosphorylation of the IκB kinase (IKK) complex (33). Phosphorylation of the IKK complex results in the ubiquitination and subsequent degradation of the NF-κB inhibitor, IκB, thus releasing NF-kB and allowing it to translocate to the nucleus whereby it induces pro-inflammatory gene expression (34).

The phenomenon of oxidative stress-driven inflammaging is described as the “oxi-inflamm-aging” theory. First proposed by De La Fuente et al., it is extensively discussed in the literature in the context of age-related inflammatory diseases (35–38). In brief, this theory builds on the previously established “free-radical theory of ageing” which proposed that ageing is a consequence of damage accumulation caused by the cumulative oxidation of biomolecules, due to the increased presence of free radicals and ROS (39). The ‘oxi-inflamm-aging’ theory further adds that the immune response is greatly implicated in this process, with increased oxidative stress shown to sustain activation of immune cells, thus leading to increased low-grade systemic inflammation and elevated risk of disease (35). In CVD for example, excessive ROS production is known to promote oxidation of low-density lipids, which largely comprise the arterial plaque (40). Ox-LDL in turn promotes recruitment of monocytes and macrophages which become polarized towards a classically activated inflammatory state, and further contribute to plaque formation (41). Classically activated macrophages produce additional ROS, which amplifies the inflammatory response in the arterial wall and progresses the disease (42). Following myocardial infarction, ROS greatly impair effective healing of the cardiac tissue, promoting dysfunction of endothelial cells and disrupting immune cell-mediated tissue repair processes. As a result, extensive fibrosis is associated with increased levels of oxidative stress and reduced cardiac function in patients (43, 44).

Similar disruptions to tissue repair and regeneration are observed in OA due to oxidative stress (45, 46). For example, excessive levels of ROS have been shown to reduce the sensitivity of chondrocytes to growth factors in articular cartilage, thus impeding tissue repair (47). Furthermore, ROS inhibits collagen synthesis in an IL-1 dependent manner, demonstrating the dual role of inflammation and oxidative stress in this disease (48). In neurodegenerative conditions, increased ROS promotes apoptosis of neural cells, and thus the release of DAMPs which drive local inflammation and further accumulation of amyloid-beta plaques in Alzheimer’s disease (AD) (49). ROS is also known to drive Tau phosphorylation in rat models of AD, which represents an additional marker of AD progression (50). Finally, in cancer, ROS are known to enhance tumorigenesis through Ras signaling, which is responsible for a number of tumorigenic processes including cell growth, proliferation and migration (51). This effect is mediated through activation of the NF-KB signaling pathway, and enhanced in inflammatory settings, highlighting the cohesion between oxidative stress and chronic inflammation in cancer progression.

## The Nrf2/HO-1 axis and oxidative stress

The clear and established links between oxidative stress and age-related disease underscore the importance of an effective antioxidant response to support healthy aging. Central to this defense mechanism is nuclear factor erythroid 2 (NF-E2)-related factor 2 (Nrf2), a redox sensitive transcription factor that induces the expression of a wide range of antioxidant and detoxification genes (52). Under homeostatic conditions, Nrf2 is bound to protein Kelch-like ECH-associated protein 1 (Keap1) in the cytoplasm. Keap1 forms part of an E3-ligase complex and promotes continuous proteasome-dependent degradation of Nrf2, thereby suppressing its activity (53, 54). Activation of Nrf2 occurs in the presence of Nrf2 activators, including ROS, which modify cysteine residues on KEAP1, allowing for the release and stabilization of Nrf2. The transcription factor can then translocate into the nucleus where it dimerizes with one of the small musculoaponeurotic fibrosarcoma (sMAF) proteins and binds to the antioxidant response element (ARE), or Maf protein recognition element (MARE), to promote expression of various cytoprotective and antioxidant genes, including *HMOX1, NQO1* and *SOD* (52, 55). Under basal conditions, sMAF proteins heterodimerize with the transcription factor BTB and CNC homology 1 (BACH1), which competes with Nrf2 for binding to the ARE site (56). However, ROS increases intracellular levels of free heme which is a ligand for Bach1. Upon heme binding, BACH1 dissociates from the ARE and is exported from the nucleus allowing Nrf2 to take its place (57, 58).

HMOX1 encodes heme oxygenase (HO-1), which together with its constitutively expressed counterpart, HO-2, catalyzes the first and rate-limiting step in the breakdown of heme, an iron-containing porphyrin that functions as a component of biological proteins such as hemoglobin and myoglobin, cytochromes, and enzymes (e.g: hem peroxidase). Heme breakdown by HO enzymes yields carbon monoxide (CO), iron, and biliverdin, which is further reduced to bilirubin by biliverdin reductase A (BVR-A). Nrf2 and BACH1 are the primary regulators of HO-1, however it is worth noting that other transcription factors such as HIF1α have been shown to regulate HO-1 expression (59). The products of heme catabolism have proven anti-inflammatory, antioxidant, and antiapoptotic activity, and given the critical link between oxidative stress and inflammation in driving disease progression, activation of the HO system has become an attractive therapeutic strategy to mitigate oxidative stress and modulate inflammatory pathways (60).

## The Nrf2-HO-1 system and inflammaging

Numerous studies in humans and mouse models have linked HO-1 deficiency to chronic inflammation (61–66). HMOX1^-/-^ mice display significantly higher production of pro-inflammatory cytokines such as IL-6, IL-12, and TNF compared to wild-type mice (62), and increased IFNβ production when challenged with LPS (67). On the other hand, HO-1 induction has shown benefit in dampening inflammation linked to inflammatory diseases such as atherosclerosis and autoimmune conditions (68, 69). The importance of HO-1 in tissue protection has also been demonstrated in patients with HO-1 genetic deficiencies that exhibit damage to the liver, kidneys, and vasculature as a result of heme and iron accumulation (70), and in murine models in which HO-1 deficiency leads to high levels of circulating heme and inflammation (71).

Recent studies have demonstrated that induction of HO-1 with hemin blocks senescence in chronic obstructive pulmonary disease lung fibroblasts by improving mitochondria function and reducing ROS levels (72). Similarly, BVR-A which reduces BV to bilirubin has been shown to protect lens epithelial cells against oxidative stress and senescence which has implications for age-related cataract (ARC) (73). Finally, it was recently demonstrated that HO-1 is required for an effective DNA damage response (DDR) and protection from senescence in multiple cells types including macrophages (74), further establishing the role of HO-1 in maintaining tissue homeostasis. This protective role was observed to be mechanistically regulated via activation of the mammalian target of rapamycin (mTOR)/S6 signaling pathway, which has already been previously acknowledged for its therapeutic benefit as both an anti-inflammatory and anti-inflammaging strategy (75).

Nrf2, has also been implicated in protection from inflammaging (see ref (76) for a detailed review). Levels of Nrf2 are decreased in aged rats and this is accompanied by increased oxidative stress (77). While most studies have focused on rodents, Nrf2 was found to be reduced during the aging of human skin fibroblasts while pharmacological activation of Nrf2 delays senescence and increases longevity in these cells (78). Furthermore, Nrf2 was found to be dysfunctional in the skeletal muscles of sedentary elderly people (79), suggesting that direct activation of Nrf2 can promote healthy aging. There is a caveat however, given that prolonged activation of Nrf-2 can actually enhance the survival of cancer cells and protect them from oxidative damage (80). Finally, excessive Nrf2 activation may drive ROS production by increasing the expression of enzymes such as NADPH oxidase (81), and thereby promote cellular senescence. Therefore, transient rather than sustained activation of Nrf2 may represent a more appropriate approach when exploring the anti-inflammaging potential of the Nrf2/HO-1 system.

## Targeting the Nrf-2/HO-1 axis to treat inflammaging

Nrf2 activators and HO-1 inducers have proven efficacy in a number of inflammatory and autoimmune disease models (82–84), as well as models of organ rejection and graft failure (85–87). Notably, in recent years, HO-1 induction has been implicated as a possible therapeutic strategy to treat infectious diseases including COVID-19 (88). Hemin-induced HO-1 was shown to mitigate cytokine storm and tissue injury in murine models of sepsis and renal damage (89), while CoPP-induced HO-1 was shown to inhibit influenza A and RSV viral replication (90). However, certain limitations surrounding the use of existing HO-1 inducers in a therapeutic context lie in their poor oral bioavailability and cytotoxicity making them unsuitable for clinical use (91). Nonetheless, investigation is ongoing into the identification of alternative, novel HO-1 inducers that could potentially mitigate current challenges to clinical translation.

In the context of inflammaging, Nrf2 activation has received greater attention thus far, particularly with respect to age-related neurodegenerative disease. This is prompted by studies such as those demonstrating that deficiency of Nrf2 is accompanied by increased neuroinflammation in the MPTP mouse model (92), while Nrf2^-/-^ mice exhibit increased amyloid-beta accumulation and significantly worse cognitive impairment when compared to control mice in murine models of AD (93). Nrf2 activators can act either through inhibition of the repressor proteins Keap1 or BACH1, or through direct regulation of the MARE/ARE domain. For example, the antioxidant tert-Butylhydroquinone (t-BHQ) binds to Keap1 cysteine residues, preventing already bound-Nrf2 from being targeted for ubiquitination, and allowing newly synthesized Nrf2 to bypass Keap1 and translocate to the nucleus (94). Administration of tBHQ has been shown to inhibit LPS-induced NLPR3 inflammasome activation as well as LPS-induced activation of microglia in mice (95). CDDO-methyl-amide (CDDO-MA) also induces Nrf2 activity through modification of cysteine residues in the Broad complex, Tramtrack, and Bric-à-Brac (BTB) domain of Keap1, which in turn prevents effective binding of Nrf2. The electrophile dimethyl fumarate (DMF), an established Nrf2 activator and FDA approved therapy for multiple sclerosis (MS), also operates in a similar manner (96). Both DMF and CDDO-MA have proven neuroprotective effects in models of age-related neurodegenerative disease. DMF has been shown to improve cognitive function in a mouse model of combined tauopathy and amyloidopathy which is representative of AD-associated pathology, while CDDO-MA, was found to reduce plaque formation and improve memory in a transgenic mouse model of AD (97, 98). In the case of Parkinson’s disease (PD), DMF was also found to protect from synucleinopathy, further highlighting the therapeutic benefit of this compound in age-related neurodegenerative conditions beyond MS (99, 100). It is worth noting however the limitation of this therapeutic, as DMF can bind non-specifically to other protein thiol groups, thus creating unwanted alterations to protein function (101). Other Keap1 inhibitors include synthetic triterpenoid compounds such as Omaveloxolone, which has recently been approved by the FDA for the treatment of Friedreich’s ataxia, a rare inherited neurogenerative disorder (102). There are significant side effects associated with these alkylating agents and more specific Keap1 inhibitors are therefore sought after. For example, a number of direct peptide-based inhibitors have been developed to inhibit the Keap1/Nrf2 protein-protein interaction (103). Cell permeability can be a limitation in this case, however it is possible that this can be overcome with the use of protein-like polymers (PLP), which consist of Keap1 binding peptides bound to a synthetic lipophilic polymer backbone (104). Recent *in vitro* studies revealed that treatment of HepG2 cells with these types of conjugated peptide results in a significant increase in ARE activity (104), however further *in vivo* exploration is required to fully established their therapeutic potential.

In addition to Keap1 inhibition, it may be possible to therapeutically promote Nrf2 activation via BACH1 inhibition (105). For example, N-(2-(2-hydroxyethoxy)ethyl)-1-methyl-2-((6-(trifluoromethyl)benzo[d]thiazol-2-yl)amino)-1H-benzo[d]imidazole-5-carboxamide (HPPE) is a non-electrophilic substituted benzimidazole which interacts with the heme-binding site of Bach1, thus preventing binding to the ARE/MARE site (101). It was recently demonstrated that HPPE administration has protective effects in the MPTP mouse model of PD (106). This was accompanied by a significant increase in Nrf2 activity and HO-1 expression, and the effects observed were more potent than hemin (106), a synthetic mimic of heme which is not suitable for clinical use.

Similar therapeutic effects can also be seen upon direct induction of HO-1 with CoPP which reduces T cell infiltration into the CNS in the EAE mouse model of MS (107), while expression of the enzyme itself is reduced in PBMC from MS patients (108). Furthermore, the heme metabolites, CO and bilirubin, produced as a result of heme breakdown by HO-1, improved disease outcome when administered at low doses in the EAE model (109–111). Both transgenic over expression of HO-1 and pharmacological induction protected from A*β* toxicity in neuronal cells and while Nrf2-dependent induction of HO-1 is largely considered cytoprotective, it has been postulated that Nrf2-independent HO-1 induction, for example by AP-1 or NF-κB, may exert cytotoxic effects in the CNS (112), hence the mode as well as duration of HO-1 induction needs to be considered when tailoring compounds for clinical use, particularly in the CNS where iron accumulation as a result of heme breakdown can contribute further to neurodegeneration via ROS production.

While more clinical trials are required, herbal medicine compounds such as resveratrol and curcumin have reported neuroprotective effects in AD, however both exhibit poor bioavailability, and there are likely to be further complications associated with delivery to the CNS. On the other hand, the Nrf-2 activator/HO-1 inducer, sulforaphane, which is found in certain cruciferous vegetables, exhibits good oral bioavailability and blood brain barrier (BBB) permeability (113), and has shown efficacy in neurodegenerative disease models including AD, PD and MS (114). The flavonoid, pinocembrin, can also traverse the BBB and was found to significantly reduce MPP^+^-induced neurotoxicity, ROS production, and neuron cell death via HO-1 induction by Nrf2 (115, 116). Therefore, sulforaphane and pinocembrin represent naturally derived compounds that could serve as add-on therapies to treat/slow age-related neurodegenerative diseases.

In addition to neurodegenerative disease, several studies have also suggested a protective role for Nrf2-HO-1 signaling in OA (117–119). Increased expression of HO-1 in murine models, due to Bach1 deficiency, significantly reduces disease severity in aged mice (118). Nrf2-HO-1 signaling not only combats inflammation, reducing the production of cytokines such as TNF, IL-1β, IL-6, and IL-18 (83), but also exhibits a protective effect in the OA tissue, preventing mitochondrial damage and apoptosis of OA chondrocytes as a result of exposure to inflammatory cytokines (120). Furthermore, HO-1 induction can simultaneously promote the regeneration of damaged cartilage by enhancing the expression of anabolic factors such as IGF-1, proteoglycan, and COL2A1 in chondrocytes (121). Similar effects are observed in CVD, where preclinical murine models reveal Nrf2-HO-1 signaling to also exhibit a dual anti-inflammatory and cytoprotective function. Induction of HO-1 by CoPP significantly improved cardiac function and decreased infarct size in diabetic mice subjected to myocardial infarction. Reduced inflammation characterized by a reduction in plasma levels of TNF was also observed in response to CoPP treatment post MI, while HO-1 induction also increased the activity of the Akt pro-survival pathway in cardiomyocytes (122). Further examples of Nrf2 activators/HO-1 inducers, their mechanism of action (if known) and therapeutic benefits in age-related disease, are summarized in **Table 1**.

**Table 1:** Targeting the Nrf2 HO-1 axis in age-related disease.

Nrf2 activator/HO-1 inducer	Target	Effect	Species	Disease relevance	Reference
tBHQ	Nrf2 via Keap1 inhibition	Inhibited activation of NLRP3 inflammasome. Reduced activation of microglia.	Mouse	Alzheimer’s Disease	(124)
CDDO-MA	Nrf2 via Keap1 inhibition	Reduced plaque formation. Improved memory.	Mouse	Alzheimer’s Disease	(98)
DMF	Nrf2 via Keap1 inhibition	Improved cognitive function. Protective in MPTP mouse model.	Mouse	Alzheimer’s Disease Parkinson’s disease	(97, 99, 100)
HPPE	Nrf2 via Bach1 inhibition	Protective in MPTP mouse model. Reduced activation of microglia and inflammation.	Mouse	Parkinson’s disease	(106)
CoPP	HO-1	Protective in MI mouse model. Improved cardiac function. Reduced expression of TNF.	Mouse	Cardiovascular disease	(122)
Resveratrol	Nrf2/HO-1	Reduced TNF and NO production in murine BV2 microglial cells. Improved memory in rat AD model. Reduced inflammation in rat OA model.	Mouse Rat	Alzheimer’s Disease Osteoarthritis	(83, 125, 126)
Sulforaphane	Nrf2 via Keap1 inhibition	Reduced Amyloid- beta aggregation. Improved memory in murine AD model.	Mouse	Alzheimer’s Disease	(127)
Pinocembrin	Nrf2/HO-1	Inhibits neuronal cell death. Protective in MPTP mouse model.	Mouse	Alzheimer’s disease Parkinson’s disease	(115, 116)
ND-13 peptide	Nrf2	Reduced accumulation of intracellular ROS. Attenuates MPTP toxicity *in vivo*.	Human mouse	Parkinson’s disease	(128)
Genistein	Nrf2/HO-1	Reduced NO, COX-2, MMP-1, MMP-3, MMP-13 in IL-1β-treated chondrocytes.	Human	Osteoarthritis	(129)
Myricetin	Nrf2/HO-1 via PI3K/Akt activation	Reduced TNF, IL-6, NO, iNOS, PGE2 in human chondrocytes. Protective in murine destabilisation meniscus model (DMM).	Human Mouse	Osteoarthritis	(130)
Cardamonin	Nrf2/HO-1	Reduced TNF, IL-1β, IL-18, IL-6 production. Reduced expression of fibrotic markers TGFβ1 and α-smooth muscle actin.	Mouse	Cardiovascular disease	(131)
Curcumin	Nrf2/HO-1	Reduced levels of MCP-1 and macrophage infiltration post-injury.	Rat	Cardiovascular disease	(132)

## Concluding remarks

**Figure 1:**
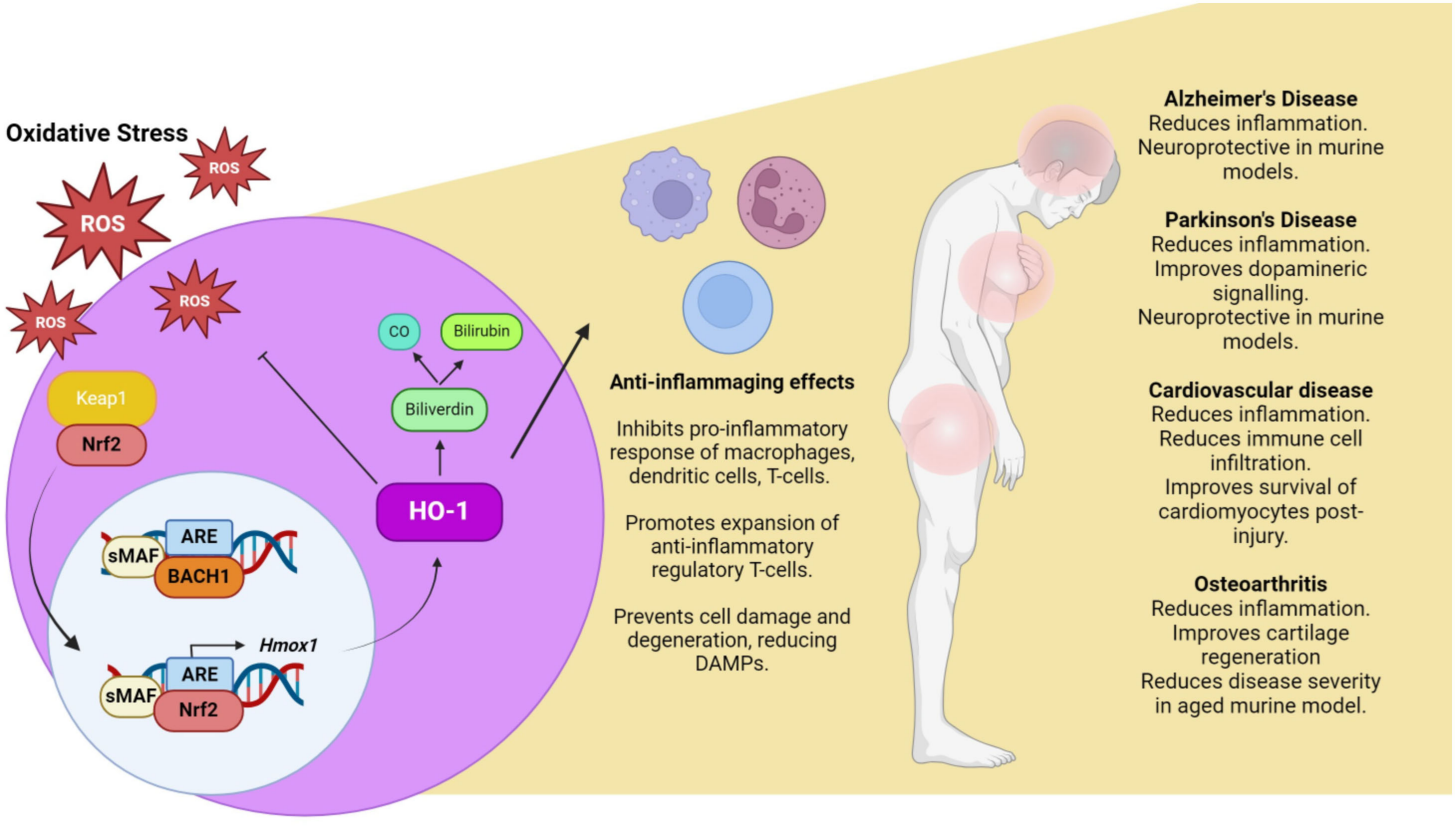
Oxidative stress, inflammaging and the Nrf2/HO-1 axis. Under conditions of oxidative stress, ROS react with cysteine residues on KEAP1, allowing for the translocation of Nrf2 to the nucleus, where it dimerises with sMAF and out competes Bach1 for binding to antioxidant response element (ARE). This promotes the expression of a number of antioxidant genes including HO-1. The primary function of HO-1 is to breakdown heme, generating CO and biliverdin which is then further reduced to bilirubin. The products of HO-1 catabolism have proven anti-inflammatory/anti-oxidant properties, highlighting the Nrf2/HO-1 axis as a potential therapeutic target to combat inflammaging. Created in BioRender. O'rourke, S. (2024).

While further study is required, strong evidence is emerging to suggest that modulation of the Nrf2 HO-1 system may have a positive impact on inflammaging and age-related inflammatory disease (**Figure 1**). However, there still exists certain limitations surrounding the use of existing Nrf2 activators/HO-1 inducers in the clinic. It is unlikely that sustained activation of Nrf2/HO-1 is a viable solution, as a number of reports have found that prolonged activation of this axis leads to numerous undesirable consequences, such as cytotoxicity as well as dysregulation of hematopoietic regeneration (112, 123). As mentioned previously, many naturally occurring HO-1 also inducers exhibit poor bioavailability, hence the mode as well as duration of Nrf2 activation/HO-1 induction needs to be considered when tailoring compounds for clinical use. Finally, prolonged Nrf2 activation is reported to promote cancer cell growth and survival hence potential drug candidates should also be screened for possible tumorigenic effects. Nonetheless, timely and controlled activation of this pathway in specific cell types represents a promising avenue to reduce age-related oxidative stress and inflammaging. Further study will determine how best to approach this and may open up a new therapeutic avenue as the demand grows for the treatment of age-related diseases.
